# Study protocol: SPARCLE – a multi-centre European study of the relationship of environment to participation and quality of life in children with cerebral palsy

**DOI:** 10.1186/1471-2458-6-105

**Published:** 2006-04-25

**Authors:** Allan Colver

**Affiliations:** 1 Professor of Community Child Health, Northumbria Healthcare NHS Trust and School of Clinical Medical Sciences (Child Health), University of Newcastle upon Tyne, Sir James Spence Institute, Royal Victoria Infirmary, Queen Victoria Road, Newcastle upon Tyne NE1 4LP, UK

## Abstract

**Background:**

SPARCLE is a nine-centre European epidemiological research study examining the relationship of participation and quality of life to impairment and environment (physical, social and attitudinal) in 8–12 year old children with cerebral palsy. Concepts are adopted from the International Classification of Functioning, Disability and Health which bridges the medical and social models of disability.

**Methods/Design:**

A cross sectional study of children with cerebral palsy sampled from total population databases in 9 European regions. Children were visited by research associates in each country who had been trained together. The main instruments used were KIDSCREEN, Life-H, Strength and Difficulties Questionnaire, Parenting Stress Index. A measure of environment was developed within the study. All instruments were translated according to international guidelines. The potential for bias due to non response and missing data will be examined. After initial analysis using multivariate regression of how the data captured by each instrument relate to impairment and socio-economic characteristics, relationships between the latent traits captured by the instruments will then be analysed using structural equation modelling.

**Discussion:**

This study is original in its methods by directly engaging children themselves, ensuring those with learning or communication difficulty are not excluded, and by studying in quantitative terms the crucial outcomes of participation and quality of life.

Specification and publication of this protocol prior to analysis, which is not common in epidemiology but well established for randomised controlled trials and systematic reviews, should avoid the pitfalls of data dredging and post hoc analyses.

## Background

The International Classification of Functioning, Disability and Health (ICF) [1] takes account of the social model of disability [2] which considers disability to result from the interaction between individuals and their environment rather than being a characteristic of the individual. The ICF introduces Environmental Factors into its classification, defining them as the physical, social and attitudinal environment in which people live and conduct their lives. Theses factors include arrangements for educational provision, social attitudes and norms, legislation on access to buildings, anti-discrimination legislation, transport design, rehabilitation, therapeutic services and assistive technology.

Aspects of the environment in European countries are adapted to respond to the needs of the disabled child and their family; some being consistent across a country but varying considerably between countries. For instance, about one third of countries must provide wheelchair access to trains by law; whilst a different one third must allow such access to cinemas. There are also wide variations in financial benefits and availability of specialised services. The rationale for such arrangements arises from theoretical standpoints and from qualitative studies [3-5], but it is not known which are effective because they have rarely been evaluated against well-defined outcomes. For example the comprehensive review "What works in services for families with a disabled child?" [6] could only examine what parents thought they needed because of the lack of research-based evidence on the effectiveness of interventions. There is a need for high quality scientific evidence to inform national and European Union (EU) policies for disabled children.

This study uses two outcomes to assess effectiveness of provision for children with disability: an objective measure of what the child does – Participation; and a subjective measure of how the child feels – Quality of Life (QoL).

The ICF introduced the concept of Participation, defining it as involvement in life situations, with the following domains: learning and applying knowledge, general tasks and demands, communication, mobility, self-care, domestic life, interpersonal interactions and relationships, major life areas and community, social and civic life. The concept of Participation replaced that of handicap, introduced by the ICF's predecessor the ICIDH [7], which was rarely used in childhood because it was too medical and did not take sufficient account of the social construction of disability [8].

The WHO defines QoL as an individual's perception of their position in life in the context of the culture and value systems in which they live, and in relation to their goals, expectations, standards and concerns [9]. In particular it is a person's self reported, subjective account of their quality of life across a number of dimensions. This is sometimes called health related quality of life (HRQOL) with health referring to the WHO definition as a state of complete physical, mental and social wellbeing [10]. HRQOL also distinguishes it from concepts which include factors external to the person such as poverty, living in a police state, and wider environmental factors such as pollution [11]. HRQOL is also distinct from concepts such as functional disability or handicap [12] which a decade or more ago were sometimes called QoL.

Research on children's QoL used to rely on their parents' or other proxys' perceptions, but it is now appreciated that the child's view should, where possible, be sought directly rather than being inferred from proxy reports. Measurement of QoL in children has lagged behind that in adults because of concern about the reliability of children's self reports, and the different values they place on particular health states as compared to adults [13,14]. Early work developed measures for specific diseases such as cancer [15] and asthma [16] for the purpose of contributing to the evaluation of medical interventions, but there is now the need for generic measures which allow comparisons not only across children with different diseases states but also across children with and without impairments.

### Overall purpose and hypothesis

Although a number of qualitative studies asking disabled children and their families to report their experience have yielded important insights into the lives and views of such children [4,5], larger populations of disabled children now need to be studied quantitatively to determine how and why participation and quality of life vary between children with comparable severity of impairment. We are studying children with cerebral palsy (CP) because:

• CP is the commonest cause of significant motor impairment, occurring in 1 in 500 births or 10,000 new cases a year in the EU prior to recent enlargement.

• Children with CP often have impairments of learning, hearing, vision, communication and epilepsy in addition to their motor ones and so are representative of the wider population of disabled children.

• Children with CP are a group with relatively stable impairment where Participation and QoL will be influenced by social and educational environmental factors as well as by medical interventions.

• Adults with CP are disadvantaged in social relationships and employment [17,18] and children are disadvantaged in education, social relationships and employment prospects [3,19].

• There are population registers of such children, reducing the risk of bias in selection of cases.

• Children aged 8–12 years were targeted because they are much less studied than preschool children, can self-report their QoL and have not entered the adolescent stage where additional factors operate.

The principal hypothesis addressed is that children with similar severity of impairment will experience variable outcomes, in terms of Participation and QoL, due to variation in Environmental Factors.

As some Environmental Factors will be consistent across a country but vary between countries, the study should allow the identification of those Environmental Factors which, if improved, will yield the greatest benefits for children and their families. Such knowledge will be invaluable in informing EU policy in the health, educational and social sectors.

Pre-specification of randomised controlled trials [20] and systematic reviews [21] increases confidence in conclusions reached but is not common practice in epidemiology. The aims of this paper are to specify objectives, hypotheses and methods prior to data analysis; and so increase confidence in the validity of the conclusions by avoiding the pitfalls of data dredging and *post hoc *analyses [22].

## Methods and design

### Ethics

Ethics approval was sought from the appropriate body in each country. The different regulations are described in an internal paper but one essential requirement in each country was that there should be special information sheets and consent forms for children. In order to fulfil this requirement, focus group work was undertaken in one of the centres, Northern Ireland, with parents and children to develop such materials.

### Population

The names of the children eligible for the study are recorded on CP registers covering all children in defined geographical areas, each child classified for impairment by type and extent. The registers share the same definition of CP [23], include all severities of CP and make every effort to be comprehensive by receiving notifications from multiple sources. Fourteen centres with such registers form a collaboration [23], and eight of these centres joined the present study (Table 1): North England, West Sweden, Northern Ireland, South East France, South West Ireland, East Denmark, Central Italy, South West France. A further centre in North West Germany joined the study after the start and followed all study protocols; however its sample could not be drawn from a validated population database and was constructed from referrals from clinicians, statutory and voluntary bodies working with children with cerebral palsy in a defined geographic area.

In order to maximise numbers in the smaller centres, children with dates of birth between 31/7/1991 and 01/04/97 inclusive (i.e. between 7 years 3 months and 12 years 11 months on 1^st ^July 2004) were included with the oldest being approached immediately and the youngest not until near their eighth birthday.

As milder cerebral palsy is more common, in the centres with sufficient numbers (1, 6) we sought similar numbers of children at each severity level by grouping children into four strata by walking ability and selecting a random sample from each stratum. Centres 2 and 3 selected a random sample of children in the stratum of best walking ability and approached all children in the remaining strata. In the smaller centres (4, 5, 7, 8) all children were approached.

The target sample size in each stratum of walking ability was estimated from power calculations using Life-H scores of children with CP [24]. A change of 2 in mean Life-H score allows the most participatory children with severe impairment to achieve what many children with only moderately severe impairment achieve. It was estimated that 30 cases were needed in each stratum to detect a difference in mean scores of 2 and that the European collaboration could provide such numbers.

### Training and visits

Training of the research associates from the different countries together was essential for quality control. They had to speak sufficient English to be able to take advantage of the training workshops, which included instruction in administering questionnaires, engaging children, disability issues and the rationale for the study. Following this, each research associate carried out up to five pilot visits in their own country; they all then met together again for the second training workshop at which difficulties and dilemmas were discussed and clear decisions made about how these should be resolved.

Children and families were therefore visited by researchers trained both to administer questionnaires to parents and to engage children for completion of their questionnaires. The visits usually took place in the child's home, lasting between 90 and 120 minutes. When the parents allowed it and the child agreed, the child completed the QoL instrument in private with the researcher.

Children with communication difficulties in addition to their CP were included by ensuring assistance from a parent, teacher or therapist as necessary. It was realised that some children with learning difficulties might not be able to report their QoL. A literature review was undertaken [25] to establish how to assess whether such children could self report their quality of life and how to interpret proxy responses from a parent or other person who knew the child well. The review showed that studies of assessment of QoL in children have never addressed the issue of self-report in children with intellectual impairment. Based on this review, we introduced a procedure to assess ability to respond to QoL questionnaires and in particular children's understanding and use of Likert scale The procedure, described by Cummins [26] for adults with intellectual impairment, was adapted for children [27]. The child is asked to order 3 real wooden cubes according to size; then to match each cube to a picture scale; then to mention things they dislike, like a bit and like a lot; then to match these to the picture scale. If successful the procedure is attempted with 5 levels.

### Instruments

The chosen instruments are listed in Table 2. An internal paper, available from the author, details how these instruments are scored and their psychometric properties.

QoL was captured by KIDSCREEN [28] which has been developed over the last four years by a multi-centre European research group. Its important features are that: it was developed from children's focus groups, it is designed for child or parent report, scaling has been developed using item response theory and shown to be consistent with a Rasch model, it possesses cross cultural validity in the translations already available and normative data are available from the general child population in each country.

Participation was captured by Life-H [24]. This instrument was developed in Quebec from a strong theoretical framework aligned with ICF [1], is well validated and has been used before in populations of children with CP.

Psychological and behavioural factors within the child were captured by the Strength and Difficulties Questionnaire SDQ [29]. This is also a well validated questionnaire and all necessary translations were already available. We used the parent report version.

Stress and functioning within the family were captured by the Parenting Stress Index [30], a well validated instrument. The Life Stress Scale of the index was used to measure the context of the parents' stress. Normative data were available from all countries in SPARCLE except Denmark – and a condition of permission to use the instrument was that normative data should be collected in Denmark following translation.

We also used the Child Health Questionnaire CHQ-PF50 [31]. This is a well validated questionnaire which has been used in many child studies, including some of children with CP. Normative data are available. It duplicates some data captured by other instruments in SPARCLE and is somewhat dated, being derived from adult questionnaires. However we decided to use it in case newer questionnaires did not function as expected and because it has a number of domains which could be used to assess construct validity of other instruments used in SPARCLE.

The numbers of children in the study, the defined impairments and socio-economic characteristics and the distribution of these factors are shown in Table 1.

To describe the environmental factors operating at a national or macro level, a review and search for relevant information was carried out by a social scientist. The results are available in an internal paper and include factors such as arrangements for educational provision, social attitudes, legislation on access to buildings, anti-discrimination legislation, transport design, rehabilitation, therapeutic services and assistive technology in the participating countries.

To describe the direct experience of environmental factors relevant to a particular child and family and the degree to which the environment helps or hinders, an instrument, the European Child Environment Questionnaire (ECEQ), was developed. Initial work included a literature review [32], and a qualitative study [33], followed by focus group work [34] in each country participating in SPARCLE. A domain structure was postulated on the basis of this qualitative work and is currently being verified using explanatory item response models [35].

As the Life-H instrument for participation does not capture frequency of participation for discretionary activities such as leisure, an instrument – the Frequency of Participation Questionnaire (FPQ) – was developed to capture frequency for those Life-H items for which it was meaningful. This was also administered to children in the general population in the relevant age range in each country to provide comparative normative data.

Translation of instruments followed international guidelines [36,37], with two forward translations and one backward one. A small amount of cultural adjustment was also needed concerning sporting activities, school types and parental socio-economic status. Table 2 details the translations undertaken.

### Data quality

To ensure data quality, questionnaires were photocopied and sent to the co-ordinating centre where data were entered into an Access database. This was a continuous process from which centres received immediate feedback about omissions, ambivalent entries or inconsistency in their returns so that corrections could be submitted. The database was programmed to assist accurate data entry, and further validations were performed after exporting into a statistical package (STATA 8). After 100 records, histograms of the distribution of responses for every question in every questionnaire were generated and inspected for face validity. Questionnaires from the first 500 children were double entered by an external company. As errors were less than 1% in all questionnaires except that about background factors, only this questionnaire was double entered for the remainder of the children.

### Statistical methods

Data quality will be assessed, in particular: heterogeneity between centres, the potential for bias due to non-response and the extent and pattern of missing data. We will compare the proportions of children in these categories in each centre with the overall proportion using a relative risk function [38]. We will assess whether non-response was related to age, gender and level of impairment and whether any such associations can explain the differences in response rates between centres [39].

The psychometric properties of the data captured by the instruments in Table 2 will be compared with the properties reported by their developers. The following will be undertaken where relevant:

• Cronbach alpha for internal consistency, within countries and overall;

• Convergent/divergent validity by considering correlations between domains and/or correlation with a similar instrument;

• Confirmatory analysis to evaluate whether the underlying factors in the data are consistent with those which have been reported in reference populations.

A separate paper will be submitted for publication around each instrument describing these validations, summarising the data, comparing with similar studies and using multivariate linear or logistic regression to assess how scores on each domain vary with level and type of impairment and socio-economic characteristics. Due to sampling within strata defined by walking ability by some centres, all analyses will be adjusted for gross motor function. A further paper will compare parents' and children's assessments of the child's quality of life and explore the factors influencing the differences; and another paper explores whether severity of impairment can be represented by a single or possibly two latent variables.

As North West Germany did not ascertain their cases from a population-based register, all analyses will include a sensitivity analysis with and without this centre.

We are also very interested in how the concepts captured by the instruments vary between countries. We are still not sure whether to compare countries using fixed effect or random effect (equivalent to multi-level) models to allow for any residual correlation of responses within centres that is not explained by the recorded variables [40]. We will then explore whether residual differences between countries can be explained by factors operating at national level, as determined by the work of the social scientist described earlier.

Following these initial assessments of how the data for each instrument relate to the child's impairment and the family's socio-demographic characteristics, relationships between the latent traits captured by the instruments will be analysed using structural equation modelling [41].

These models will address the principal hypotheses – that Environment affects QoL and Participation – in separate models for the various domains of the child's experience (e.g. physical, social and attitudinal environment at home and school). They will be built up iteratively, at first considering only Environment and QoL while stratifying by impairment, but subsequently introducing the direct and indirect influences of socio-economic factors, the child's personality and behaviour, and parental stress as possible modifying factors. Finally, we shall explore a model which includes both QoL and Participation.

Whilst many factors may influence both outcomes, our primary interest focuses on the effects of environment after allowing for other factors. By setting out in advance the relationships which may be causative, we expect that structural equation modelling will allow confirmation or rejection of those postulated causal links.

## Discussion

SPARCLE is a large, multi-centre study carried out in nine European centres. The results will be widely disseminated and it is essential that findings will be credible to academics in disability studies, social science, child health, psychology, to politicians and to developers of social, health and educational policy at national and European levels. This paper is an essential part of this process in that it sets out, prior to analysis, the background to the project, the concepts involved, the methods adopted and the analyses which will be undertaken.

There are a number of aspects to this study which have rarely been brought together in studies of children and disability. Wherever possible, the self-report of the child of their QoL has been obtained and used as the subjective outcome. However where children can not self report, they have been included but their QoL is reported by a proxy. The concept of Participation, introduced in the ICF and fully embraced by the European Commission's Research Frameworks is the other central measured outcome. The concept of the physical, social and attitudinal environment introduced by the ICF is also a crucial element of this study and an instrument to capture its experience by child and family has been specially developed. The environment at the national level is also being sought systematically and set out by a social scientist; and will allow the study to investigate the relationship of differences in environment between countries to differences in outcome between countries.

The study has a number of methodological strengths. It uses a network of population based registers of all children with CP living in defined geographic areas, from which either all children were included or children were randomly sampled; this allows thorough analysis of potential bias as registration data were known for those children whose families could not be contacted or who declined to take part. The questionnaires were translated according to international guidelines; the research associates who visited the children and families were trained together and adopted common procedures with common understanding; special attention was paid to data quality and completeness.

We recognise possible limitations to the study. Some of the development of the environmental questionnaire, the ECEQ, is taking place within the study because a large data set was necessary for the final stages of scaling and item reduction. However we also have access to a further data set of children where the ECEQ had been completed in children with a variety of different severe impairments which has allowed some independent validations to be undertaken (Forsyth R, Personal Communication). We deliberately chose a QoL instrument suitable for all children not just disabled children but it will be important to investigate how valid this is, especially in the children with severe intellectual or motor impairment. Kidscreen is already known to behave consistently in the general population across different European countries but we will examine whether there is differential item functioning between disabled children and the general population.

## Competing interests

The author(s) declares he has no competing interest

## Authors' contributions

The author is co-ordinator of the SPARCLE project, compiled the original grant application to the European Commission and has written this manuscript.

## Pre-publication history

The pre-publication history for this paper can be accessed here:



## Figures and Tables

**Table 1:** Included children by centre, impairment and socio-economic characteristics

**Centre**	**No. of children**	**No. *(%) *of males**	**Median age in years (IQR)**
			
North England, UK	116	74 *(64)*	10.5 (2.5)
West Sweden	83	44 *(53)*	10.5 (2.1)
Northern Ireland, UK	102	63 *(62)*	10.3 (2.3)
South East France	67	38 *(57)*	10.6 (2.4)
South West Ireland	98	53 *(54)*	10.2 (2.3)
East Denmark	115	73 *(63)*	10.5 (2.9)
Central Italy	85	47 *(55)*	10.4 (2.5)
South West France	77	48 *(62)*	10.3 (3.1)
North West Germany	75	43 *(57)*	10.1 (2.8)
**TOTAL**	**818**		
**Classification**	**Category**		**No.*(%) *of children**
	
**Impairment**			
*Cerebral palsy type*			
	Unilateral spastic		279 *(34)*
	Bilateral spastic		423 *(52)*
	Dyskinetic		86 *(11)*
	Ataxic		29 *(4)*
	(Missing)		1 *(0.1)*
			
*IQ*			
	>70		385 *(47)*
	50–70		186 *(23)*
	<50		242 *(30)*
	(Missing)		5 *(0.6)*
			
*Gross Motor Function Classification System [42]*			
	GMFCS 1		257 *(31)*
	GMFCS 2		164 *(20)*
	GMFCS 3		139 *(17)*
	GMFCS 4		113 *(14)*
	GMFCS 5		145 *(18)*
	(Missing)		0 *(0)*
			
*Bimanual Fine Motor Function [43]*			
	BFMF 1		281 *(34)*
	BFMF 2		205 *(25)*
	BFMF 3		131 *(16)*
	BFMF 4		91 *(11)*
	BFMF 5		110 *(13)*
	(Missing)		0 *(0)*
			
*Vision*			
	Blind or no useful vision		59 *(7)*
	Not above		759 *(93)*
	(Missing)		0 *(0)*
			
*Hearing*			
	>70 decibels in better ear		799 *(2)*
	Not above		18 *(98)*
	(Missing)		1 *(0.1)*
			
*Seizures*			
	No seizures, no medication		576 *(70)*
	No seizures, medication		74 *(9)*
	Seizures<1 a month		63 *(8)*
	Seizures>1 a month and <1 a week		47 *(6)*
	Seizures>1 a week		57 *(7)*
	(Missing)		1 *(0.1)*
			
*Communication*			
	Normal		463 *(57)*
	Difficulty but uses speech		133 *(16)*
	Uses non speech for formal communication		98 *(12)*
	No formal communication		123 *(15)*
	(Missing)		1 *(0.1)*
			
*Feeding*			
	No problems		583 *(71)*
	Feeds orally with difficulty		176 *(22)*
	Partial or complete non oral feeding		58 *(7)*
	(Missing)		1 *(0.1)*

**Schooling**			
	Mainstream		316 *(39)*
	Split mainstream and unit		110 *(13)*
	Unit in mainstream		68 *(8)*
	Special school		310 *(38)*
	(Missing)		14 *(1.7)*

**Socio-economic characteristics **(algorithm if two parents/carers)			
			
*Best educational level*			
	High: Entry requirement for University or above		396 *(48)*
	Low: Below University requirement		415 *(51)*
	(Missing)		7 *(1)*
			
*Income based on occupation and employment*			
	Full time professional/managerial		226 *(28)*
	Full time trade		402 *(15)*
	Part time		48 *(35)*
	Neither works		141 *(24)*
	(Missing)		1 *(0.1)*
			
*Area of living*			
	Big city		120 *(15)*
	Suburbs of big city		123 *(15)*
	Town		286 *(35)*
	Village		195 *(24)*
	Countryside		91 *(11)*
	(Missing)		3 (0.4)

**Table 2:** Instruments

**Instrument**	**Reference**	**Captures**	**Domains**	**Type of variable**	**Translations required**
Life-H	[24]	Child's Participation	Communication Personal care Around the house Feeding Mobility Fitness Responsibility Recreation Education Community Life Interpersonal relations	Dependent. Primary outcome	Danish, French, German, Italian, Swedish
Frequency of Participation Questionnaire (FPQ)	Being developed within the study	Frequency of participation where measurement is meaningful and the participation is discretionary		Dependent. Primary outcome	Danish, French, German, Italian, Swedish
Kidscreen	[28]	Child's Quality of Life	Psychological Emotion Social support Home life Self perception Autonomy School Social acceptance Finance Physical well-being	Dependent. Primary outcome	Danish, Italian Minor adaptations required for professional and proxy versions in all languages
European Child Environment Questionnaire (ECEQ)	Being developed within the study	Extent to which environment facilitates or hinders participation	Physical Social Attitudinal	Explanatory	Danish, French, German, Italian, Swedish
Strength and Difficulties Questionnaire (SDQ)	[29]	Child's behaviour	Emotional symptoms Conduct problems Hyperactivity Peer problems Prosocial behaviour	Explanatory	None
Parenting Stress Index Short Form (PSI-SF)	[30]	Parental stress	Parental distress Parent-child dysfunctional interaction Difficult child PSI Life stress scale	Explanatory	Danish French for Life Stress Scale items only
Child Health Questionnaire (CHQ)	[31]	General child health	Global health Physical functioning Social emotional Social physical Bodily pain Behaviour Global behaviour Mental health Self esteem General health perceptions Change in health Parental impact – emotional Parental impact – time Family activities Family cohesion	Primarily included to validate other instruments	None
Impairment	Descriptive	Child's Impairments	Type of cerebral palsy Gross motor function Upper limb function Intelligence Hearing Vision Seizures Communication Feeding	Explanatory	Danish, French, German, Italian, Swedish
Background child and family factors	Descriptive		Age Sex School Siblings Partner status Parental academic qualifications Parental employment Parental occupation	Explanatory	Danish, French, German, Italian, Swedish
